# Lack of association between prior or concurrent malignancies and overall survival in gastroesophageal cancer: evidence from a large European single-center cohort

**DOI:** 10.1007/s12094-025-04036-3

**Published:** 2025-08-29

**Authors:** Hannah C. Puhr, Luzia Berchtold, Linda Zingerle, Martin Korpan, Julia M. Berger, Gerd Jomrich, Reza Asari, Sebastian F. Schoppmann, Gerald W. Prager, Elisabeth S. Bergen, Anna S. Berghoff, Matthias Preusser, Aysegül Ilhan-Mutlu

**Affiliations:** 1 Department of Medicine I, Division of Oncology, Medical University of Vienna, Waehringer Guertel 18-20, 1090 Vienna, Austria; 2 Christian Doppler Laboratory for Personalized Immunotherapy, Medical University of Vienna, Waehringer Guertel 18-20, 1090 Vienna, Austria; 3 Institute of Medical Statistics, Center for Medical Data Science, Medical University of Vienna, Waehringer Guertel 18-20, 1090 Vienna, Austria; 4 Department of Surgery, Medical University of Vienna, Waehringer Guertel 18-20, 1090 Vienna, Austria

**Keywords:** Gastric cancer, Esophageal cancer, Other malignancies, Survivorship

## Abstract

**Background:**

History of malignant disease is a common exclusion criterion in clinical cancer trials, yet data on the impact of cancer survivorship on outcome in gastroesophageal cancer patients are scarce.

**Methods:**

Retrospective association analyses of self-reported prior or concurrent malignancies with patient characteristics, tumor characteristics, symptoms and overall survival (OS) were performed in 1491 gastroesophageal cancers patients treated between 01/01/2000 and 31/12/2021 at the Medical University of Vienna.

**Results:**

Of 1491 patients 255 (18%) had other primary cancer diagnoses, of which 185 (73%) occurred before, 52 (20%) at the same time as and 18 (7%) both before and at the same time as gastroesophageal cancer diagnosis. 205 (80%) patients had one, 43 (17%) had 2 and 7 (3%) had 3 other malignancies. History of other malignancies was associated with older age (p < 0.0001), squamous cell histology (p = 0.018), less aggressive localized tumor stages (p = 0.037) and fewer acid reflux (p = 0.011). There was neither an association between history of other primary malignancies nor the number of other cancer entities and OS (p = 0.47; p = 0.43).

**Conclusion:**

Self-reported history of other malignant diseases is frequent in a real-life European gastroesophageal cancer cohort and was not statistically significantly associated with outcome, but rather with older age and squamous cell histology. Our data emphasize that cancer survivors should not be categorically excluded from clinical cancer trials due to fear of dismal prognosis. Prospective research is warranted to improve eligibility for this subgroup.

**Supplementary Information:**

The online version contains supplementary material available at 10.1007/s12094-025-04036-3.

## Background

As therapeutic strategies evolve, outcome and survival of cancer patients improves, while risk factors for the development of malignancies cumulate with advanced age. These include non-modifiable factors such as inherited cancer syndromes like Lynch syndrome and or CDH1 mutations, and modifiable exposures such as obesity, diet, Helicobacter pylori infection, chronic gastroesophageal reflux, smoking, and heavy alcohol use [1]. Many of these factors play a role not only in the development of the first malignancy but also in increasing the risk of subsequent cancers later in life.

Thus, patients often suffer from multiple malignant diseases throughout their life and cancer survivors are known to be at higher risk for cancer development than the general population [2–4]. However, prior or concurrent malignant diseases—either altogether or within 24 months before trial initiation—are frequent exclusion criteria in clinical trials, as these patients are often believed to have worse outcome [5]. Thereby, overly strict eligibility criteria might limit access to novel drugs for cancer survivors and create discrepancies between trial populations and real-world cohorts. Especially in cancer entities with limited treatment options and devastating outcome statistics, such as gastroesophageal cancer, execution and implementation of clinical trials for a broad population is crucial [6].

Gastroesophageal cancer is a major contributor to global disease burden. Although incidence rates decline in general, there are specific subgroups especially in Western countries that show increasing incidence rates [7]. Yet, data on frequency, characteristics and prognosis of gastroesophageal cancer patients with prior or concurrent other malignancies are scarce.

Thus, the rationale of this manuscript is to provide detailed insights in self-reported cumulation of cancerous diseases in individuals with gastroesophageal cancer and investigate whether there are prognostic differences between patients with and without history of prior or concurrent malignancies that justify the categorical exclusion of cancer survivors from clinical trials.

## Methods

### Patient and data recruitment

This analysis included patients aged ≥ 18 years, who were diagnosed and/or treated for pathologically confirmed gastroesophageal cancer between 01/01/2000 and 31/12/2021 at the Medical University of Vienna.

Concerning tumor genetics, data of programmed cell death ligand 1 (PD-L1), human epidermal growth factor receptor 2 (HER2) and mismatch repair deficiency (dMMR) were included in this analysis where available. Definition of PD-L1 positivity was based on combined positive score (CPS) ≥ 1 and/or tumor proportion score (TPS) ≥ 1%, which is routinely performed at our hospital since 2020. Definition of HER2 positivity was based on immunohistochemical staining +  +  + or immunohistochemical staining +  + with HER2 gene amplification confirmed by fluorescence in situ hybridization (FISH) using the CEP17/HER2 ratio, which has been routinely performed since 2012. MMR status was assessed by immunohistochemical staining for MLH1, PMS2, MSH2, and MSH6 and has been performed routinely since 2020. Cases with loss of expression of one or more proteins were classified as dMMR.

All patients were treated with the best available treatment according to current treatment guidelines at the time of diagnosis.

Retrospective evaluation of clinical data, including patient characteristics, tumor characteristics, symptoms, therapeutic strategies as well as data on self-reported other malignant diseases, was performed by structured electronical chart review and then collected and stored in a FilemakerPro® version 18 database (Claris International Inc., Cupertino, CA, USA). Standardized history taking ensured accuracy of data collection. History of smoking was defined as a cumulative tobacco exposure of ≥ 5 pack-years. History of alcohol consumption was defined as a documented history of harmful alcohol use, alcohol dependence, or consistent alcohol consumption exceeding 14 standard drinks per week for men or 7 for women.

Regulatory restrictions in Austria did not allow documentation of information on patients’ genetics and therefore this information is not included in this analysis.

The time of diagnosis of other primary oncological diseases was defined as follows: Before gastroesophageal cancer diagnosis: Cancer had to be fully healed (no signs of active disease in laboratory or/and imaging analysis) more than 6 months before diagnosis of gastroesophageal cancer. At the same time as gastroesophageal cancer diagnosis: Other primary cancer was diagnosed either within 6 months before or after diagnosis of gastroesophageal cancer, or gastroesophageal cancer was diagnosed while treatment for other primary cancer was still ongoing. Patients with multiple cancer diagnoses, that occurred both before and at the same time as gastroesophageal cancer diagnosis, were declared as such.

As the American Society of Clinical Oncology (ASCO)–Friends of Cancer Research recommended to broaden eligibility criteria by also including patients with prior oncological diseases, who completed therapy 24 months before registration as well as patients with concurrent tumors that are clinically stable and do not require tumor-directed treatment [6], we also investigated whether the prior malignancies that occurred within 24 months before gastroesophageal cancer diagnosis excluding in-situ carcinomas and basaliomas had an impact on the overall survival (OS).

Patients’ death was registered according to hospital chart data and/or public data provided by “Statistik Austria”. Patients with missing dates of death at the time of data cut-off were censored at the date that they were last known to be alive. OS was defined as the time from initial gastroesophageal cancer diagnosis to patient’s death or last follow-up date.

This retrospective analysis of patient data was in line with the ethical standards of the responsible committee on human experimentation and with the Helsinki Declaration of 1964 and later versions. The ethics committee of the Medical University of Vienna approved this study (reference number: 1600/2021).

### Statistical analysis

Clinical data underwent descriptive analysis, utilizing metrics like the median, range, and interquartile range (IQR) for continuous variables. Categorical variables were characterized by absolute numbers, percentages, and the count of missing values. Differences in categorical and continuous data were assessed using the Chi-square test and Wilcoxon rank-sum test, respectively.

Overall survival (OS) was analyzed using the log-rank test, which compares survival distributions between groups defined by nominal variables. Survival probabilities over time were estimated and graphically presented using Kaplan–Meier curves to illustrate differences between patient subgroups. We additionally stratified patients by histological subtype and stage at initial diagnosis. Variables that were significantly associated with OS in univariate analyses (p < 0.05) were subsequently entered into a multivariate model using Cox proportional hazards regression to identify independent prognostic factors.

Statistical significance was attributed to two-tailed p-values < 0.05.

Due to the hypothesis generating design of the studies no correction for multiple testing was implemented (8). The statistical analyses were carried out using R software (version 4.2.3; R Foundation for Statistical Computing, Vienna, Austria) via the RStudio integrated development environment (Posit Software, PBC, Boston, MA, USA).

## Results

### Patient and tumor characteristics

In total, 1491 patients with gastroesophageal cancer were treated at the Medical University of Vienna between 01/01/2000 and 31/12/2021. Concerning patient characteristics, 70% of patients (n = 1043) were men and median age at first diagnosis of gastroesophageal cancer was 64 years (range: 23–92, IQR: 55–72). When looking at behavior associated with cancer development, median BMI was 24.61 (range: 13.6–57.0, IQR: 22.0–27.6), 186 (14%) had a recorded history of alcohol consumption and 805 (59%) recorded history of smoking (median 40 packyears, range: 2–165, IQR: 24–55). The date of data cut-off was 2023 November 1 st, at which point 1156 (78%) patients were already deceased. Median overall survival (OS) from cancer diagnosis to death was 21.2 months in the overall cohort (95%CI 19.7–22.5).

Concerning symptoms at first diagnosis, 1125 (80%) patients experienced dyspepsia, 840 (60%) dysphagia, 717 (52%) weight loss, 304 (24%) abdominal pain, 192 (21%) gastrointestinal bleeding or ulceration, 234 (17%) frailty, 199 (16%) acid reflux and 161 (13%) nausea.

In respect to tumor characteristics, 1200 (80%) patients had histologically proven adenocarcinoma (AC). Considering tumor location, 555 (37%) had gastric, 482 (32%) gastroesophageal junction and 454 (31%) esophageal primary tumors. The majority of tumors were classified as locally advanced (stage 2 + 3: 756 (51%)), while 545 (36%) had metastatic (stage 4) and 190 (13%) patients had localized (stage 1) disease at first diagnosis.

More detailed information on patient characteristics, tumor characteristics, symptoms at first diagnosis and therapeutic strategies is available in Supplementary Tables 1, 2, 3, 4, respectively.

### Other primary malignancies

In 1443 patients (97%) data on other primary tumors at first diagnosis was available. There were 1188 (82%) patients without another cancer diagnosis besides their gastroesophageal cancer. 255 (18%) had other primary cancer diagnoses, of which 185 (73%) occurred before gastroesophageal cancer diagnosis, 52 (20%) at the same time as gastroesophageal cancer diagnosis and 18 patients (7%) had other primary tumors both before and at the same time of gastroesophageal cancer diagnosis.

205 (80%) patients had one other tumor entity, 43 (17%) patients had 2 other cancers and 7 (3%) patients had 3 other primary cancers.

For patients with other cancers “before gastroesophageal cancer diagnosis”, the median time between diagnosis of other primary tumor and first diagnosis of gastroesophageal cancer was 101 months before gastroesophageal cancer (min: 590 months before, max: 8.8 months before). The median time of cancers classified to have occurred “at the time of gastroesophageal cancer diagnosis” was 0.1 months before gastroesophageal cancer (min: 130 months before (ongoing malignant disease), max: 5 months after).

**Fig. 1:**
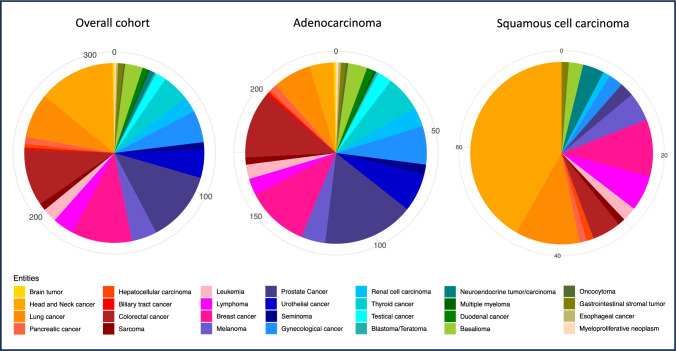
Distribution of other malignant entities in a large Western cohort of gastroesophageal cancer patients (left) as well as in adenocarcinoma (middle) and squamous carcinoma (right) subgroups

Concerning tumor entities, the most common other oncological diseases were head and neck cancer (n = 43, 14%), prostate cancer (n = 40, 13%), breast cancer (n = 34, 11%), colorectal cancer (n = 32, 10%) and lung cancer (n = 25, 8%). All frequencies of other cancer entities are shown in Table 1 and Fig. 1 for the overall cohort as well as for AC and squamous cell carcinoma (SCC) subgroups.

**Table 1:** Tumor entities of secondary oncological diseases in a cohort of gastroesophageal cancer patients

	overall		adeno		SCC	
Tumor entity	n	%	n	%	n	%
Head and neck cancer	43	14	10	4	33	42
Prostate cancer	40	13	38	16	2	3
Breast cancer	34	11	26	11	8	10
Colorectal cancer	32	10	28	12	4	5
Lung cancer	25	8	16	7	9	11
Gynecological cancer	18	6	16	7	2	3
Urothelial cancer	16	5	16	7		
Thyroid cancer	15	5	15	6		
Melanoma	14	4	10	4	4	5
Lymphoma	12	4	7	3	5	6
Basalioma	10	3	8	3	2	3
Renal cell carcinoma	9	3	8	3	1	1
Leukemia	8	3	6	3	2	3
Testical cancer	6	2	6	3		
Pancreatic cancer	4	1	3	1	1	1
Sarcoma	4	1	3	1	1	1
Seminoma	4	1	4	2		
Neuroendocrine tumor/carcinoma	3	1			3	4
Duodenal cancer	3	1	3	1		
Gastrointestinal stromal tumor	3	1	2	1	1	1
Hepatocellular carcinoma	2	1	1	< 1	1	1
Brain tumor	1	< 1	1	< 1		
Biliary tract cancer	1	< 1	1	< 1		
Blastoma/Teratoma	1	< 1	1	< 1		
Multiple myeloma	1	< 1	1	< 1		
Oncocytoma	1	< 1	1	< 1		
Esophageal cancer	1	< 1	1	< 1		
Myeloproliferative neoplasm	1	< 1	1	< 1

### Association of other primary malignancies with patient and tumor characteristics

A total of 4 (2%) patients were aged less or equal to 45 years, 87 (34%) were between 46–64 years and 164 (64%) were 65 years or older (p < 0.0001). The median age of patients with other cancer diagnoses was 68 years (range: 39–88, IQR: 61–74).

Furthermore, histological subtype was also associated with other primary malignancies (p = 0.018). While in patients without other tumors only 19% had SCC, in patients with prior or concurrent malignancies 25% were SCC. In addition, patients with other malignancies showed less stage 3 tumors (35% versus 27%, p = 0.037) and fewer acid reflux (17% versus 10%, p = 0.011).

All associations of patient and tumor characteristics with other primary tumors are shown in Table 2 and Table 3.

**Table 2:** Association of other secondary diseases with patient and tumor characteristics

	Other oncological disease		
	negative (n = 1188)	positive (n = 255)	p value***
Sex*			0.98
Male	830 (70%)	178 (70%)	
Female	358 (30%)	77 (30%)	
Age**			< 0.001
Median (IQR)	63 (54, 71)	68 (61, 74)	
Age groups*			< 0.001
≤ 45 years	101 (8.5%)	4 (1.6%)	
46–64 years	553 (47%)	87 (34%)	
≥ 65 years	534 (45%)	164 (64%)	
BMI**			0.16
Median (IQR)	24.4 (21.9, 27.5)	25.1 (22.0, 27.8)	
Missing	248	43	
BMI*			0.27
Underweight	47 (5.0%)	11 (5.2%)	
Normal weight	486 (52%)	94 (44%)	
Overweight	285 (30%)	74 (35%)	
Obese	122 (13%)	33 (16%)	
Missing	248	43	
History of smoking*			0.91
No	459 (41%)	100 (42%)	
Abuse	649 (59%)	139 (58%)	
Missing	80	16	
History of alcohol consumption*			0.10
No	489 (45%)	117 (49%)	
Moderate	444 (41%)	99 (41%)	
Abuse	162 (15%)	23 (9.6%)	
Missing	93	16	
Histological subtype*			0.018
AC	967 (81%)	191 (75%)	
SCC	221 (19%)	64 (25%)	
Tumor location*			0.16
GEJ	398 (34%)	70 (27%)	
Stomach	433 (36%)	99 (39%)	
Esphagus	357 (30%)	86 (34%)	
Stage*			0.037
1	144 (12%)	43 (17%)	
2	194 (16%)	43 (17%)	
3	425 (36%)	70 (27%)	
4	425 (36%)	99 (39%)	
Lauren classification*			0.36
Intestinal	230 (45%)	50 (51%)	
Diffuse	251 (49%)	45 (46%)	
Mixed	30 (5.9%)	3 (3.1%)	
Missing	677	157	
Signet ring cells*			0.056
Yes	357 (30%)	62 (24%)	
No	820 (70%)	193 (76%)	
Missing	11	0	
Helicobacter infection*			0.33
Yes	337 (44%)	63 (39%)	
No	436 (56%)	97 (61%)	
Missing	415	95	
MMR*			0.25
dMMR	8 (4.3%)	4 (9.1%)	
pMMR	176 (96%)	40 (91%)	
Missing	1004	211	
HER2*			0.88
Positive	101 (21%)	23 (22%)	
Negative	370 (79%)	81 (78%)	
Missing	717	151	
PD-L1*			0.62
Positive	93 (66%)	26 (70%)	
Negative	48 (34%)	11 (30%)	
Missing	1047	218	
Family history in general*			0.52
Positive	433 (42%)	85 (40%)	
Negative	595 (58%)	129 (60%)	
Missing	160	41	
Family history gatroesophageal cancer*			0.93
Positive	118 (11%)	25 (12%)	
Negative	910 (89%)	189 (88%)	
Missing	160	41	

^*^n (%)

^**^median (IQR)

^***^Chi-square and Wilcoxon-rank test as appropriate

**Table 3:** Association of secondary oncological disease with symptoms at initial diagnosis

	Other oncological diseases		
	negative (n = 1188)	positive (n = 255)	p value
Dysphagia			0.30
No	436 (39%)	103 (43%)	
Yes	679 (61%)	138 (57%)	
Missing	73	14	
Dyspepsia			0.40
No	219 (20%)	54 (22%)	
Yes	904 (80%)	193 (78%)	
Missing	65	8	
Acid reflux			0.011
No	843 (83%)	196 (90%)	
Yes	173 (17%)	22 (10%)	
Missing	172	37	
Abdominal pain			0.35
No	780 (77%)	161 (74%)	
Yes	235 (23%)	57 (26%)	
Missing	173	37	
Nausea			0.36
No	890 (88%)	186 (85%)	
Yes	126 (12%)	32 (15%)	
Missing	172	37	
Weight loss			0.28
No	520 (47%)	121 (51%)	
Yes	581 (53%)	116 (49%)	
Missing	87	18	
GI bleeding			0.18
No	873 (79%)	179 (77%)	
Yes – ulceration	88 (8.0%)	27 (12%)	
Yes – active bleeding	145 (13%)	27 (12%)	
Missing	82	22	
Frailty			0.47
No	922 (83%)	191 (81%)	
Yes	186 (17%)	44 (19%)	
Missing	80	20	

### Survival analyses

**Fig. 2:**
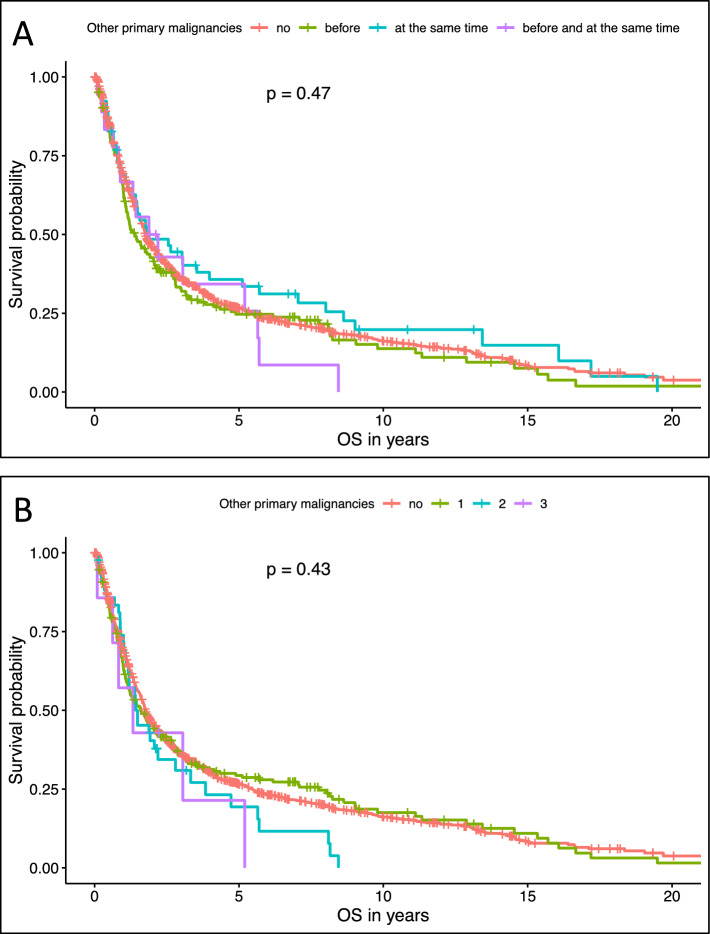
Overall survival of patients (A) with and without other oncological diseases and (B) with no, one, two or three other primary cancers prior or concurrent to the time of gastroesophageal cancer diagnosis

Self-reported history of other primary malignancies at diagnosis of gastroesophageal cancer was not statistically significantly associated with OS, as demonstrated in Fig. 2. Patients without other malignancies (n = 1188) had a median OS of 21.4 months (95%CI 20.2–23.5), while those with a history of malignancies diagnosed before the gastroesophageal cancer (n = 185) had a median OS of 16.9 months (95% CI: 13.8–24.8). Patients diagnosed with another malignancy concurrently with gastroesophageal cancer (n = 52) showed a median OS of 22.5 months (95% CI: 17.3–62.3), and those with malignancies both before and at the time of diagnosis (n = 18) had a median OS of 24.8 months (95% CI: 10.8–NA). These differences were not statistically significant (p = 0.47, see Fig. 2A).

Similarly, the number of other malignancies reported by patients was not significantly associated with OS. Patients without any other malignancies (n = 1188) had a median OS of 21.4 months (95% CI: 20.2–23.5), compared to 19.4 months (95% CI: 14.7–27.5) for those with one additional malignancy (n = 205), 18.1 months (95% CI: 14.2–34.1) for those with two (n = 43), and 16.2 months (95% CI: 7.6–NA) for those with three other malignancies (n = 7). This trend was also not statistically significant (p = 0.43, see Fig. 2B).

In respect to histological and stage subgroups, there was also no difference concerning OS in patients with or without other oncological diseases (AC: p = 0.64; SCC: p = 0.84; stage 1: p = 0.19; stage 2: p = 0.88; stage 3: p = 0.98; stage 4: p = 0.068), which is visualized in Supplementary Figs. 1 and 2, respectively. In the stage 4 subgroup, median OS did not differ significantly between patients with and without other malignancies (p = 0.068), however there was a tendency for longer survival in patients who had another oncological disease before and at the same time of cancer diagnosis. Median OS was 10.90 months (95% CI: 9.4–12.2) for patients with no other malignancy (n = 425), 10.50 months (95% CI: 7.3–12.2) for those with another malignancy before gastroesophageal cancer diagnosis (n = 72), 9.25 months (95% CI: 5.0–32.1) for those with another malignancy at the same time (n = 16), and 17.40 months (95% CI: 10.1–NA) for those with malignancies both before and at the same time (n = 11).

In a further analysis focusing only on prior malignancies diagnosed within 24 months before gastroesophageal cancer diagnosis and excluding carcinoma in situ and basal cell carcinoma, no significant association with OS was identified (p = 0.72). Patients without any prior or concurrent malignancy within this timeframe (n = 1350) had a median OS of 21.2 months (95% CI: 19.7–22.6), while those with a prior malignancy within 24 months (n = 31) had a median OS of 16.9 months (95% CI: 11.9–50.4). Patients with a concurrent malignancy (n = 60) had a median OS of 31.1 months (95% CI: 17.4–68.8), and those with both prior and concurrent malignancies (n = 2) had a median OS of 33.1 months (95% CI: 2.9–NA).

In contrast, some patient and tumor characteristics, including age (p = 0.016), year of first diagnosis (p < 0.0001), BMI (p = 0.0017), histological subtype (p = 0.0093), stage (p < 0.0001), Lauren classification (p = 0.00015), Helicobacter pylori (p = 0.0084) and HER2 (p = 0.044), dysphagia (p = 0.00088), nausea (p = 0.048), frailty (p < 0.0001) and weight loss (p < 0.0001) were statistically significantly associated with OS. More detailed information is available in Supplementary Tables 1, 2, 3, respectively.

### Multivariable analysis

In the multivariate Cox proportional hazards regression model (n = 153, 108 events), higher tumor stage (HR = 2.13, 95% CI = 1.68–2.71, p < 0.001) and the presence of frailty symptoms (HR = 2.49, 95% CI = 1.49–4.15, p < 0.001) were independently associated with worse overall survival, while higher BMI (HR = 0.32, 95% CI = 0.12–0.86, p = 0.024) was associated with better prognosis. No statistically significant associations with overall survival were found for age category, year of diagnosis, Lauren classification, Helicobacter pylori infection, HER2 status, dysphagia, nausea, or weight loss after adjustment for other variables.

## Discussion

Patients with prior or concurrent malignancies are often excluded from clinical cancer trials as they are believed to have worse outcome than other cancer patients [5]. Although there are several recommendations to include cancer survivors in clinical cancer trials, i.e. from the U.S. Food and Drug Administration (FDA) [8] and from the American Society of Clinical Oncology (ASCO)–Friends of Cancer Research [6, 9], investigators are often reluctant to include these patients presumably due to fear of impairment of trial results.

Our findings of a large European real-life gastroesophageal cancer cohort directly challenge this reluctance. Although every sixth patient had prior or concurrent malignancies, there was no statistically significant association with OS. Therefore, categorically excluding this subgroup limits accrual without a valid scientific rationale. This practice creates three major problems. First, real-world patients are underrepresented, which limits the applicability of trial results to everyday clinical practice [9]. Second, these patients lose the opportunity to access potentially beneficial novel therapies available only through clinical trials. Third, reduced recruitment may jeopardize the completion and overall success of the trial [10, 11]. The relevance of this issue is underscored by similar findings in other cohorts, with reported rates of secondary cancers in 10–15% of survivors [5, 12, 13].

Concerning tumor entities that were observed in our study, the frequency of prostate, lung, breast and colorectal cancer fit the general population and are therefore to be expected. However, there was a high number of head and neck, urothelial and thyroid carcinomas in our cohort [6]. A Swedish analysis shows similar incidence rates and entities [14]. Thus, specific tumor entities might indicate the development of gastroesophageal cancer in the future and these patients might benefit from closer surveillance and increased patient’s awareness.

This is supported by the fact, that stage is still the most relevant prognostic marker in our cohort (median OS 10.8 months in stage 4 versus 86.3 months in stage 1), which also remained statistically significant in multivariate analysis. Hence, earlier diagnosis of predefined patients at risk due to closer surveillance may lead to improved survival statistics [15, 16].

Furthermore, the development of secondary cancers is mainly linked to either lifestyle or environmental risk factors, genetic susceptibility or treatment related DNA damage caused by cytotoxic drugs or radiation [17]. However, our analysis did not show any statistically relevant associations of common behavioral risk factors including BMI, history of smoking and alcohol consumption. Older age seems to be a major factor for the development of multiple cancer entities, which is comprehensible as these patient have more time to acquire premalignant and malignant DNA mutations that cause cancer [18]. In addition, cancer treatment and genetic susceptibility may also induce secondary tumors. Genetic alterations known to be associated with gastroesophageal cancer include mutations in CDH1 (E-cadherin) tumor suppressor gene, which is associated with lobular breast cancer [19, 20], and mismatch repair proteins, which is associated with colorectal cancer [21]. Yet, as neither the treatment of previous cancerous diseases nor genetic alterations are routinely documented in our hospital charts, our investigation does not include this information, which poses a major limitation. In spite of that, there was no statically significant association with family history for cancer in general or gastroesophageal cancer with prior and concurrent malignancies, which makes genetic predispositions improbable. However, patients with other malignancies had statistically significantly less acid reflux. As gastroesophageal AC in Western cohorts is often associated with reflux and Barrett’s disease, this result insinuates that in the subgroup with prior or concurrent malignancies different pathomechanisms might lead to tumor development [15, 16, 22]. Nevertheless, no definitive causality for the development of other malignant diseases can be drawn from this retrospective analysis.

However, our analysis is the first to evaluate prognostic impact of other malignancies in a large European cohort of gastroesophageal cancer, both for prior and concurrent malignancies in general as well as for prior malignancies that occurred and needed treatment within 24 months before gastroesophageal cancer diagnosis. While there are increasing numbers of case reports from patients with synchronous multiple primary tumors [23–25], data from large cohorts are scarce. An analysis performed with data from Surveillance, Epidemiology, and End Results (SEER) database, indicated that specific prior cancer subtypes might show inferior survival in gastric cancer patients. However, as this analysis incorporated patients from a very large time frame (from 1975 to 2016), these results might be biased by outdated treatment regimen [26]. The rationale behind this potential bias is supported by the results from a second analysis from the SEER database investigating patients diagnosed between 2004 and 2011, which did not show any inferiority [27]. The bias of time was minimized in our analysis by extracting information from the past 20 rather than 50 years, but still persists in our data as well.

In conclusion, our analysis shows that there is no statistically significant difference in survival outcomes between gastroesophageal cancer patients with or without prior or concurrent malignancies. Therefore, our data emphasize that cancer survivors should not be categorically excluded from clinical cancer trials due to fear of dismal prognosis. Integrating this subgroup could improve recruitment rates, enhance representativeness, and allow the generation of evidence that is more applicable to real-world patient populations. Prospective research is warranted to improve eligibility for this subgroup.

## Supplementary Information

Below is the link to the electronic supplementary material.

Supplementary Figure 1: Overall survival in adenocarcinoma patients (A) as well as in squamous cell carcinoma patients (B) with and without other primary malignancies at the time of diagnosis. Supplementary file1 (PDF 45 KB)

Supplementary Figure 2: Overall survival in patients with gastroesophagral cancer stage 1 (A), stage 2 (B), stage 3 (C) and stage 4 (D) with and without other primary malignancies at the time of diagnosis. Supplementary file2 (PDF 68 KB)

Supplementary file3 (DOCX 14 KB)

Supplementary file4 (DOCX 15 KB)

Supplementary file5 (DOCX 14 KB)

Supplementary file6 (DOCX 15 KB)

## Data Availability

The data that support the findings of this study are available from the corresponding author upon reasonable request.

## Abbreviations

OS: Overall survival
HR: Hazard ratio
CI: Confidential interval
HER2: Human epidermal growth factor receptor 2
PD-L1: Programmed cell death ligand 1
BMI: Body mass index
dMMR: Mismatch repair deficiency
CPS: Combined positive score
TPS: Tumor proportion score
IQR: Interquartile range
AC: Adenocarcinoma
SCC: Squamous cell carcinoma
FDA: U.S. food and drug administration
ASCO: American society of clinical oncology
SEER: Surveillance, epidemiology, and end results
