# Supplementary material for: Lack of association between prior or concurrent malignancies and overall survival in gastroesophageal cancer: evidence from a large European single-center cohort
Source: Clin Transl Oncol. 2025 Aug 29;28(3):942–52. doi: 10.1007/s12094-025-04036-3 (PMC12920280; doi:10.1007/s12094-025-04036-3)
Supplement: Supplementary file 1 — Supplementary Figure 1: Overall survival in adenocarcinoma patients (A) as well as in squamous cell carcinoma patients (B) with and without other primary malignancies at the time of diagnosis. Supplementary file1 (PDF 45 KB) [file 12094_2025_4036_MOESM1_ESM.pdf]

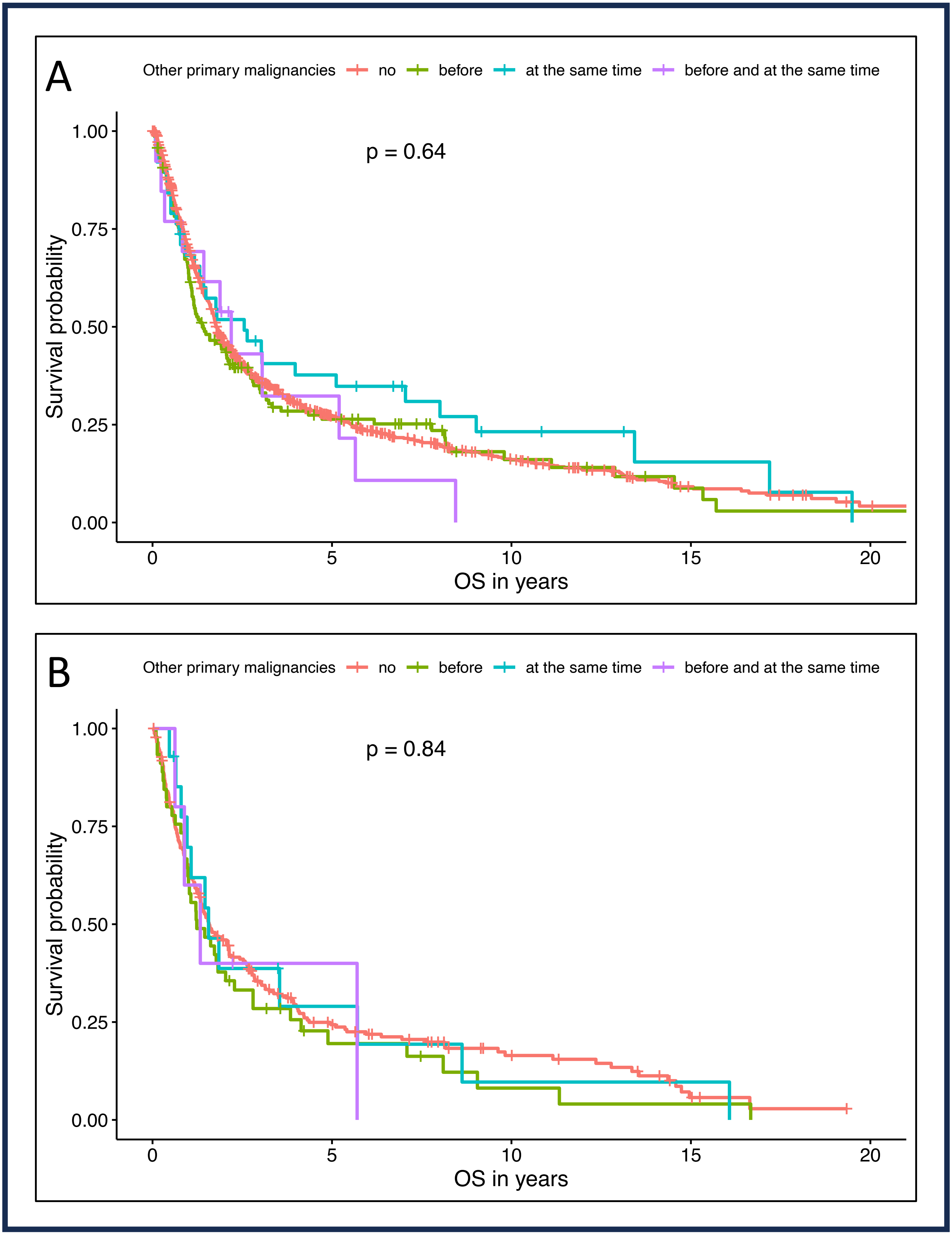
A
Other primary malignancies
no
before
at the same time
before and at the same time
p = 0.64
Survival probability
1.00
0.75
0.50
0.25
0.00
0
5
10
15
20
OS in years
B
Other primary malignancies
no
before
at the same time
before and at the same time
p = 0.84
Survival probability
1.00
0.75
0.50
0.25
0.00
0
5
10
15
20
OS in years
