# Supplementary figures and images for: Lack of association between prior or concurrent malignancies and overall survival in gastroesophageal cancer: evidence from a large European single-center cohort
Source: Clin Transl Oncol. 2025 Aug 29;28(3):942–52. doi: 10.1007/s12094-025-04036-3 (PMC12920280; doi:10.1007/s12094-025-04036-3)

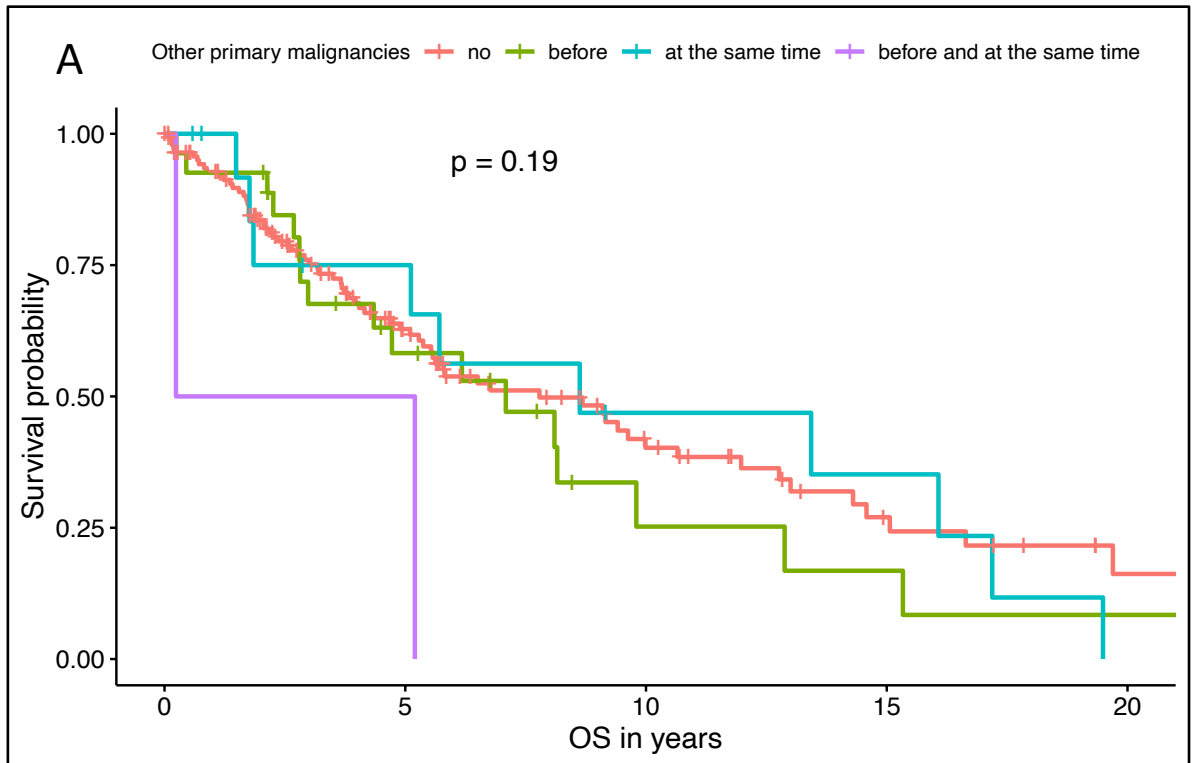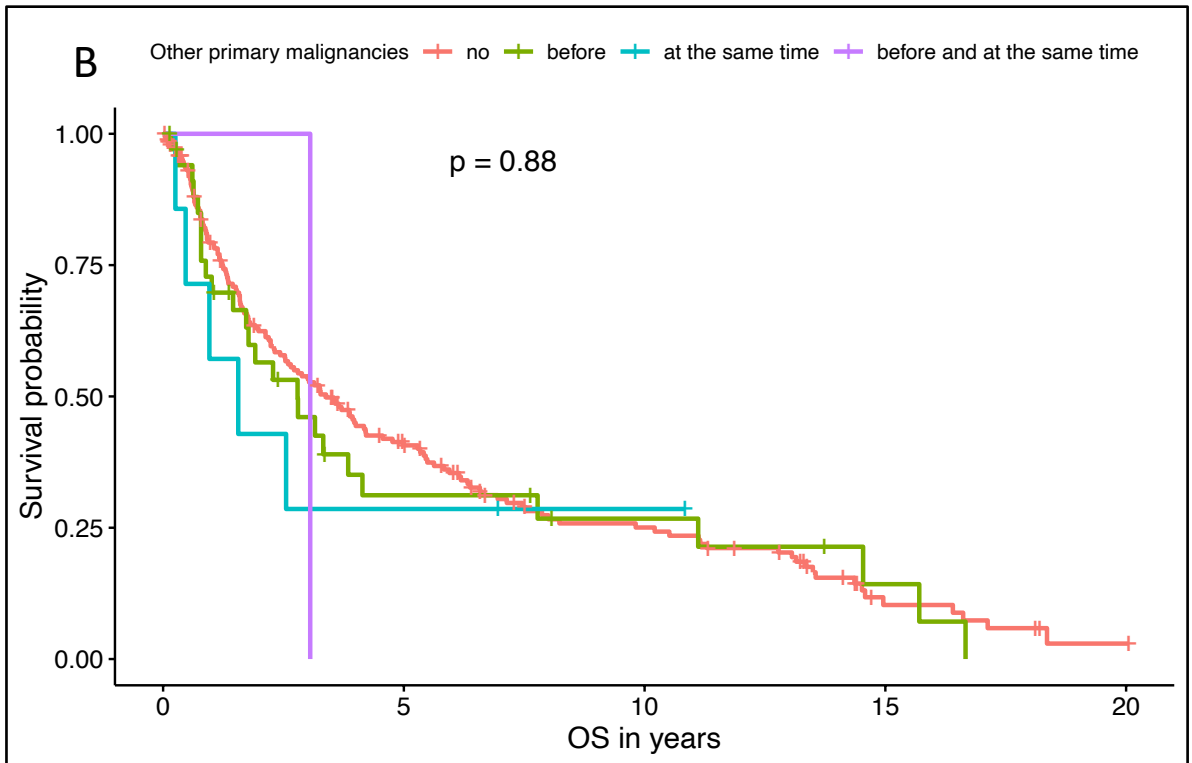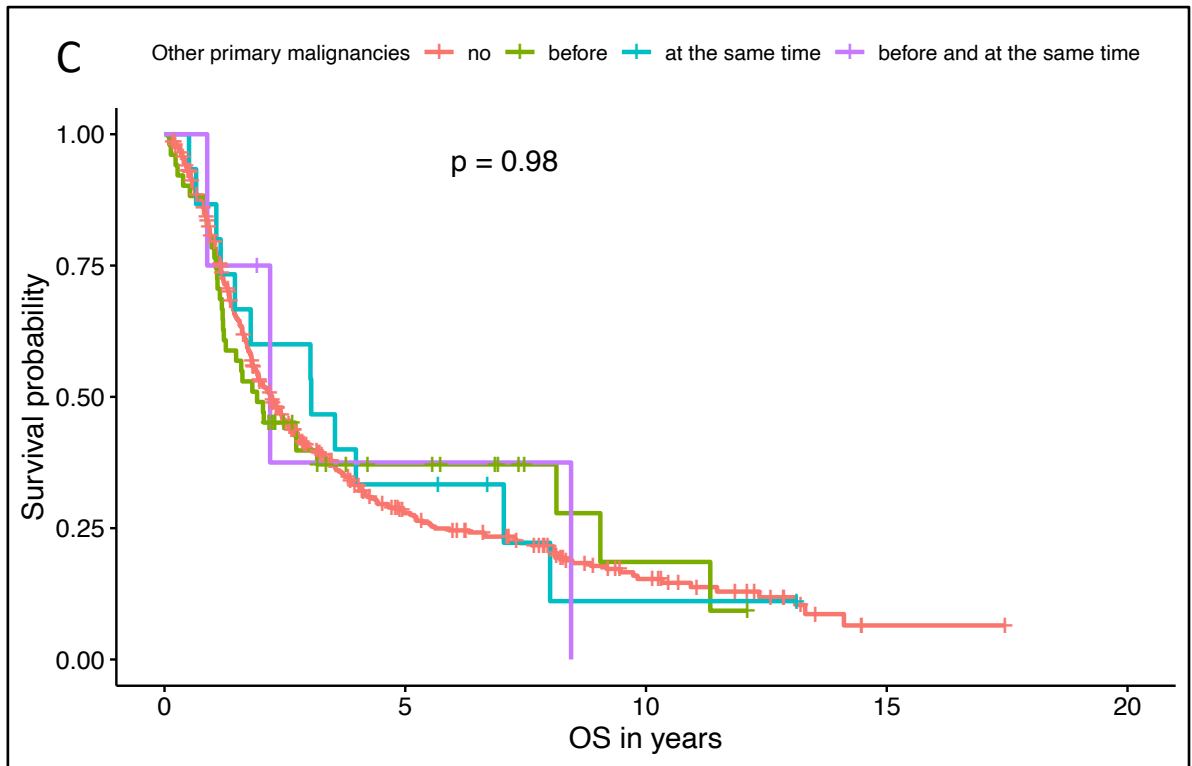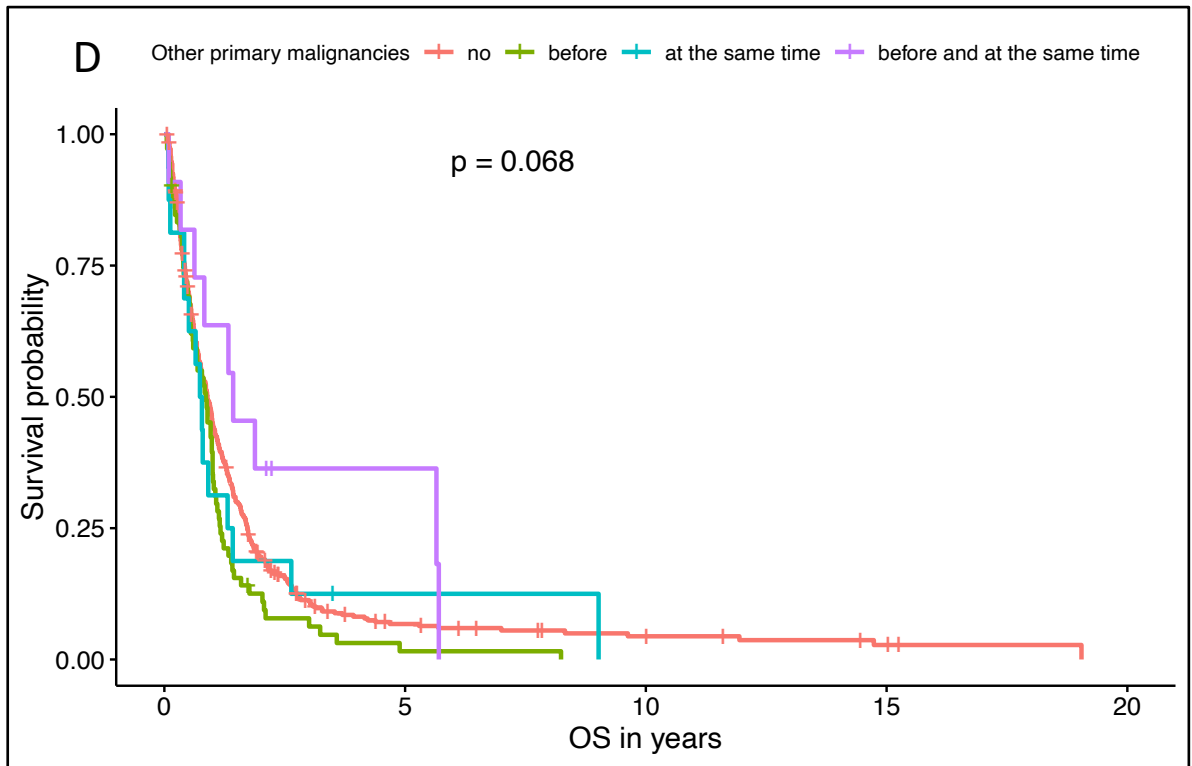

Supplement: Supplementary file 2 — Supplementary Figure 2: Overall survival in patients with gastroesophagral cancer stage 1 (A), stage 2 (B), stage 3 (C) and stage 4 (D) with and without other primary malignancies at the time of diagnosis. Supplementary file2 (PDF 68 KB) [file 12094_2025_4036_MOESM2_ESM.pdf]
