# Supplementary material for: Lack of association between prior or concurrent malignancies and overall survival in gastroesophageal cancer: evidence from a large European single-center cohort
Source: Clin Transl Oncol. 2025 Aug 29;28(3):942–52. doi: 10.1007/s12094-025-04036-3 (PMC12920280; doi:10.1007/s12094-025-04036-3)
Supplement: Supplementary file 3 — Supplementary file3 (DOCX 14 KB) [file 12094_2025_4036_MOESM3_ESM.docx]

| **Characteristics** | **Value, n (%)** | **Median OS in months (95%CI)** | **p-value** |
| --- | --- | --- | --- |
| **Sex** |  |  | p=0.61 |
| Male | 1043 ( 70 %) | 21.2 (19.4-22.8) |  |
| Female | 448 ( 30 %) | 21.2 (19.4-24.5) |  |
| **Age** |  |  | **p=0.016** |
| ≤45 | 110 ( 7 %) | 27.7 (22.0-40.8) |  |
| 46-64 | 662 ( 44 %) | 21.0 (19.1-23.5) |  |
| ≥65 | 719 ( 48 %) | 20.7 (18.0-22.7) |  |
| **Year of first diagnosis** |  |  | **p < 0.0001** |
| 2000-2005 | 298 ( 20%) | 32.3 (27.2-39.8) |  |
| 2006-2010 | 434 ( 29 %) | 16.5 (14.4-19.5) |  |
| 2011-2015 | 319 ( 21 %) | 22.0 (19.7-26.1) |  |
| 2016-2021 | 440 ( 30 %) | 20.0 (17.3-22.8) |  |
| **Body mass index** |  |  | **p=0.0017** |
| Underweight | 58 ( 5 %) | 13.3 (7.7-16.4) |  |
| Normal weight | 586 ( 50 %) | 20.9 (18.6-24.5) |  |
| Overweight | 364 ( 31 %) | 23.3 (20.7-26.6) |  |
| Obese | 159 ( 14 %) | 23.1 (18.0-40.6) |  |
| Missing data | 324 |  |  |
| **History of alcohol consumption** |  |  | p=0.057 |
| No alcohol | 614 ( 45 %) | 21.2 (19.0-23.5) |  |
| Moderate | 554 ( 41 %) | 21.5 (19.7-25.1) |  |
| Abuse | 186 ( 14 %) | 16.8 (13.8-23.3) |  |
| Missing data | 137 |  |  |
| **History of smoking** |  |  | p=0.25 |
| No nicotine abuse | 564 ( 42 %) | 20.2 (17.2-22.0) |  |
| Nicotine abuse | 805 ( 58 %) | 22.0 (20.0-25.5) |  |
| Missing data | 122 |  |  |

Supplementary table 1: Patient characteristics and their association with the overall survival (log rank test).
