# Supplementary material for: Lack of association between prior or concurrent malignancies and overall survival in gastroesophageal cancer: evidence from a large European single-center cohort
Source: Clin Transl Oncol. 2025 Aug 29;28(3):942–52. doi: 10.1007/s12094-025-04036-3 (PMC12920280; doi:10.1007/s12094-025-04036-3)
Supplement: Supplementary file 4 — Supplementary file4 (DOCX 15 KB) [file 12094_2025_4036_MOESM4_ESM.docx]

| **Characteristics** | **Value, n (%)** | **median OS in months (95%CI)** | **p-value** |
| --- | --- | --- | --- |
| **Histological subtype** |  |  | **p=0.0093** |
| Adeno | 1200 ( 80 %) | 21.5 (20.4-23.2) |  |
| SCC | 291 ( 20 %) | 19.0 (16.2-24.8) |  |
| **Tumor location** |  |  | p=0.054 |
| Stomach | 555 ( 37 %) | 21.2 (19.5-22.9) |  |
| GEJ | 482 ( 32 %) | 22.0 (19.4-26.7) |  |
| Esophagus | 545 ( 31 %) | 20.2 (16.5-24.8) |  |
| **Stages** |  |  | **p<0.0001** |
| Stage 1 | 190 ( 13 %) | 86.3 (67.4-117.2) |  |
| Stage 2 | 246 ( 16 %) | 38.2 (30.9-48.3) |  |
| Stage 3 | 510 ( 34 %) | 26.7 (23.5-30.7) |  |
| Stage 4 | 545 ( 37 %) | 10.8 (9.6-12.0) |  |
| **Lauren classification** |  |  | **p=0.00015** |
| Intestinal | 287 ( 45 %) | 26.6 (22.5-37.1) |  |
| Diffuse | 312 ( 49 %) | 19.0 (16.4-21.3) |  |
| Mixed | 34 ( 6 %) | 25.9 (14.3-40.5) |  |
| Missing | 858 |  |  |
| **Signet ring cells** |  |  | p=0.065 |
| Signet ring cells | 436 ( 30 %) | 19.6 (17.4-21.5) |  |
| No signet ring cells | 1038 ( 70 %) | 22.1 (20.7-24.9) |  |
| Missing | 17 |  |  |
| **Helicobacter pylori** |  |  | **p=0.0084** |
| H. pylori infection | 410 ( 43 %) | 24.9 (21.5-32.2) |  |
| No H. pylori infection | 548 ( 57 %) | 22.0 (20.0-25.5) |  |
| Missing | 533 |  |  |
| **Mismatch repair deficiency** |  |  | p=0.36 |
| dMMR | 12 ( 5 %) | 19.4 (10.0-NA) |  |
| pMMR | 220 ( 95 %) | 24.5 (21.2-32.4) |  |
| Missing | 1259 |  |  |
| **HER2** |  |  | **p=0.044** |
| HER2 positive | 125 ( 21 %) | 30.3 (22.2-36.9) |  |
| HER2 negative | 466 ( 79 %) | 19.8 (17.0-21.6) |  |
| Missing | 900 |  |  |
| **PD-L1** |  |  | p=0.13 |
| PDL1 positive | 120 ( 67 %) | 19.7 (17.3-28.1) |  |
| PDL1 negative | 59 ( 33 %) | 18.1 (13.4-23.6) |  |
| Missing | 1312 |  |  |

Supplementary table 2: Tumor characteristics and their association with the overall survival (log rank test).
