# Supplementary material for: Lack of association between prior or concurrent malignancies and overall survival in gastroesophageal cancer: evidence from a large European single-center cohort
Source: Clin Transl Oncol. 2025 Aug 29;28(3):942–52. doi: 10.1007/s12094-025-04036-3 (PMC12920280; doi:10.1007/s12094-025-04036-3)
Supplement: Supplementary file 5 — Supplementary file5 (DOCX 14 KB) [file 12094_2025_4036_MOESM5_ESM.docx]

| **Characteristics** | **Value, n (%)** | **Median OS in months (95%CI)** | **p-value** |
| --- | --- | --- | --- |
| **Dysphagia** |  |  | **p=0.00088** |
| No | 553 ( 40 %) | 23.3 (20.7-27.9) |  |
| Yes | 840 ( 60 %) | 19.5 (17.4-21.6) |  |
| Missing | 98 |  |  |
| **Dyspepsia** |  |  | p=0.089 |
| No | 281 ( 20 %) | 19.0 (16.5-23.6) |  |
| Yes | 1125 ( 80%) | 21.4 (20.1-23.3) |  |
| Missing | 85 |  |  |
| **Acid reflux** |  |  | p=0.7 |
| No | 1069 ( 84 %) | 21.5 (20.1-23.8) |  |
| Yes | 199 ( 16 %) | 20.1 (16.6-26.2) |  |
| Missing | 223 |  |  |
| **Abdominal pain** |  |  | p=0.73 |
| No | 963 ( 76 %) | 21.8 (20.6-24.1) |  |
| Yes | 304 ( 24%) | 19.7 (16.9-23.8) |  |
| Missing | 224 |  |  |
| **Nausea** |  |  | **p=0.048** |
| No | 1107 ( 87 %) | 21.8 (20.8-24.9) |  |
| Yes | 116 ( 13 %) | 16.0 (13.0-22.1) |  |
| Missing | 223 |  |  |
| **GI bleeding** |  |  | p=0.099 |
| No | 1081 ( 79 %) | 20.4 (18.1-21.6) |  |
| Yes – ulceration | 117 ( 8 %) | 21.3 (17.3-32.4) |  |
| Yes – active bleeding | 175 ( 13 %) | 23.1 (20.1-32.8) |  |
| Missing | 118 |  |  |
| **Frailty** |  |  | **p<0.0001** |
| No | 1142 ( 83 %) | 22.1 (20.9-25.2) |  |
| Yes | 234 ( 17 %) | 12.8 (11.3-17.1) |  |
| Missing | 115 |  |  |
| **Weight loss** |  |  | **p<0.0001** |
| No | 657 ( 48 %) | 26.2 (23.1-30.9) |  |
| Yes | 717 ( 52 %) | 16.0 (14.5-17.6) |  |
| Missing | 117 |  |  |

Supplementary table 3: Symptoms and their association with the overall survival (log rank test).
