# Supplementary material for: Lack of association between prior or concurrent malignancies and overall survival in gastroesophageal cancer: evidence from a large European single-center cohort
Source: Clin Transl Oncol. 2025 Aug 29;28(3):942–52. doi: 10.1007/s12094-025-04036-3 (PMC12920280; doi:10.1007/s12094-025-04036-3)
Supplement: Supplementary file 6 — Supplementary file6 (DOCX 15 KB) [file 12094_2025_4036_MOESM6_ESM.docx]

| Characteristics | Value, n (%) |
| --- | --- |
| Surgery |  |
| Surgical resection | 934 ( 63 %) |
| Systemic therapy |  |
| Neoadjuvant/perioperative | 366 ( 25 %) |
| Pseudoneoadjuvant^ | 13 ( 1 %) |
| Adjuvant (without neoadjuvant regimen) | 102 ( 7 %) |
| Palliative with initial curative intent | 63 ( 4 %) |
| Palliative 1^st^ line | 593 ( 40 %)* |
| Palliative 2^nd^ line | 266 ( 18 %) |
| Palliative 3^rd^ line | 94 ( 6 %) |
| Palliative 4^th^ line | 21 ( 1 %) |
| Palliative 5^th^ line | 10 ( 1 %) |
| Radiotherapy |  |
| Radiotherapy of primary tumor | 253 ( 17 %)** |
| Radiotherapy of primary tumor recurrence | 48 ( 3 %) |
| Radiotherapy of metastatic sites | 111 ( 7 %) |

Supplementary table 4: Therapeutic strategies
^neoadjuvant regimen given to oligometastatic patients in order to achieve surgical respectability.
*516 (35%) of patients received first line chemotherapy without prior systemic therapy for gastroesophageal cancer, **100 (7%) received definitive radiochemotherapy
